# Organ preservation in rectal cancer with contact x-ray brachytherapy (Papillon): a refined Swiss protocol to improve real-world feasibility

**DOI:** 10.3389/fonc.2025.1608427

**Published:** 2025-07-09

**Authors:** Cristina Picardi, Francesca Caparrotti, Nora Brunner-Schaub, Daniel Christen, Marie Fargier-Voiron, Michael Drepper, Alain Von Laufen, Michael Montemurro, Frederic Ris, Oscar Matzinger

**Affiliations:** ^1^ Department of Radiation Oncology, Swiss Medical Network, Zürich, Switzerland; ^2^ Department of Radiation Oncology, Clinique Générale Beaulieu, Swiss Medical Network, Geneva, Switzerland; ^3^ Department of Gastroenterology, Bethanien Klinik, Swiss Medical Network, Zürich, Switzerland; ^4^ Department of Surgery, Bethanien Klinik, Swiss Medical Network, Zürich, Switzerland; ^5^ Department of Radiation Oncology, Swiss Medical Network, Genolier, Switzerland; ^6^ Department of Gastroenterology, Clinique Generale Beaulieu, Geneva, Switzerland; ^7^ Medical Oncology, Clinique de Genolier, Genolier, Swiss Medical Network, Genolier, Switzerland; ^8^ Department of Visceral Surgery, Hopitaux Universitaires de Geneve, Geneva, Switzerland

**Keywords:** rectal cancer, organ preservation, radiotherapy, brachytherapy, dose escalation

## Abstract

Rectal cancer is an increasingly prevalent malignancy, with growing interest in organ preservation strategies as an alternative to radical surgery, particularly for tumors of the mid-lower rectum. Radiotherapy plays a central role in these approaches and contact X-ray brachytherapy (CXB or Papillon technique) allows for safe dose escalation directly to the tumor, enhancing local control while minimizing toxicity. This report describes the development and implementation of a refined protocol for Papillon CXB, addressing technical challenges related to patient comfort, applicator placement, and procedural accuracy. By the end of 2024, a total of 129 patients with mid-lower rectal cancer had been treated with Papillon CXB in Swiss centers. In February 2022, a refined protocol was introduced, incorporating procedural sedation (Propofol), the lateral decubitus position, and close collaboration with gastroenterologists to improve tumor visualization and precise applicator placement. This updated approach has been applied to 73 patients, mainly with curative intent and organ preservation goals. Sedation significantly improved patient comfort and reduced movement during applicator insertion. The applicator demonstrated excellent stability, even in anatomically challenging cases. The procedure proved to be straightforward, smoothly integrated into clinical workflows, and well-tolerated by patients, with high satisfaction reported by both patients and the medical team. This Swiss protocol offers a practical and patient-centered refinement of Papillon CXB. By addressing critical technical and clinical challenges, it enhances procedural feasibility and safety, ultimately supporting optimal oncological outcomes and wider adoption in rectal cancer management.

## Introduction

Rectal cancer remains a significant public health challenge, accounting for a large proportion of colorectal malignancies worldwide (1, 2). Traditionally, it has been most prevalent in older adults (over 70 years), who often have multiple comorbidities that complicate surgical management (3). Recent epidemiological trends, however, show an alarming increase in rectal cancer incidence among younger populations, with up to 25% of new cases now occurring in individuals under 50 years old (4, 5). These younger patients may face decades of post-treatment sequelae, while older patients continue to contend with surgical risks. In both groups, radical resection often leads to a permanent stoma and associated bowel dysfunction, such as Low Anterior Resection Syndrome (LARS), severely impacting quality of life (6).

Standard curative treatment for locally advanced rectal cancer typically involves a trimodality approach with neoadjuvant (chemo)radiotherapy followed by total mesorectal excision (TME), offering high rates of tumor control but often at the expense of function and long-term quality of life.

Against this backdrop, organ-preserving approaches have emerged, seeking to avoid radical surgeries and enhance patients’ long-term well-being. Habr-Gama and colleagues pioneered non-operative management for carefully selected patients achieving complete clinical response (cCR) after radiotherapy (RT) or radiochemotherapy (CRT) (7). Subsequent data from the International Watch & Wait Database (IWWD) further validated these findings and highlighted that local regrowth typically remains confined to the rectum, allowing for prompt salvage surgery (8). While these strategies initially focused on early-stage disease, the OPRA trial showed that with a total neoadjuvant therapy (TNT) concept, which intensifies systemic therapy, organ preservation could be achieved in approximately 50% of patients with more advanced (cT3–cT4) tumors (9).

Radiotherapy is central to achieving effective local control in rectal cancer (10, 11). Dose escalation improves the likelihood of cCR (12), but it is limited by the risk of toxicity to surrounding pelvic structures. Papillon contact X-ray brachytherapy (CXB) addresses this challenge by delivering a highly localized, high-dose radiation boost directly to the tumor, sparing surrounding tissues and facilitating both tumor downstaging and potential organ preservation. Multiple studies have demonstrated the benefits of Papillon CXB in enhancing organ preservation and local control in rectal cancer (12–18). Among these, the OPERA trial provided robust evidence, showing an overall organ preservation rate of 79% with CXB compared to 56% with standard external beam radiotherapy (P = 0.004). The advantage was even more pronounced in smaller tumors (<3 cm), with a preservation rate of 93% versus 54%, highlighting its effectiveness in nearly doubling long-term organ preservation in select cases (18, 19).

Although Papillon CXB is primarily associated with organ preservation strategies, it is a versatile treatment in rectal cancer. When combined with CRT, it increases sphincter preservation by approximately 30% and reduces the risk of permanent stoma (13). Moreover, although its primary role is curative, CXB is also effective as salvage therapy after local relapse, post-polypectomy, or for palliative symptom control.

Since 2015, Papillon CXB has been an integral part of rectal cancer management in Swiss Medical Network (SMN) centers in Zurich and Geneva. Early experience revealed common limitations, particularly in anatomically challenging, lower rectal tumors, related to patient discomfort and the risk of applicator slippage. To overcome these challenges, we developed a refined protocol that integrates sedation, strategic patient positioning, and collaboration with gastroenterologists. This work focuses on the implementation and procedural details of the Swiss protocol for Papillon CXB, highlighting practical adaptations to improve patient comfort and procedural precision. Building on our previously published clinical data demonstrating high rates of organ preservation (17), this paper emphasizes the technical innovations that enhance the feasibility and applicability of CXB. By sharing these refinements, we aim to support the broader adoption of this technique as a cornerstone of organ-preserving strategies in rectal cancer management.

## Patient characteristics

Between October 2015 and December 2024, a total of 129 consecutive patients with rectal adenocarcinoma were treated using Papillon CXB at two SMN centers in Zurich and Geneva. We introduced and implemented the protocol with propofol sedation in collaboration with gastroenterologists in February 2022, and by the end of 2024, 73 patients had been treated using this method.

Papillon CXB was offered in different clinical scenarios. The most common indication for contact radiotherapy is planned organ preservation. Patients were selected for this purpose if they had histologically confirmed rectal cancer (cT1–cT3), nodal status N0 or N1, and no evidence of metastatic disease (n = 50). Those with larger tumors (cT4a), >N1 nodal involvement, or synchronous metastases were evaluated on a case-by-case basis (n = 5). Other indications for Papillon CXB included salvage therapy after local relapse (n = 7), treatment following resection of a T1 polypoid tumor (n = 3), and palliative treatment (n =8).

Treatment decisions were made by a multidisciplinary tumor board, considering each patient’s surgical and oncological risks.

## Papillon CXB device and delivery

**Figure 1:**
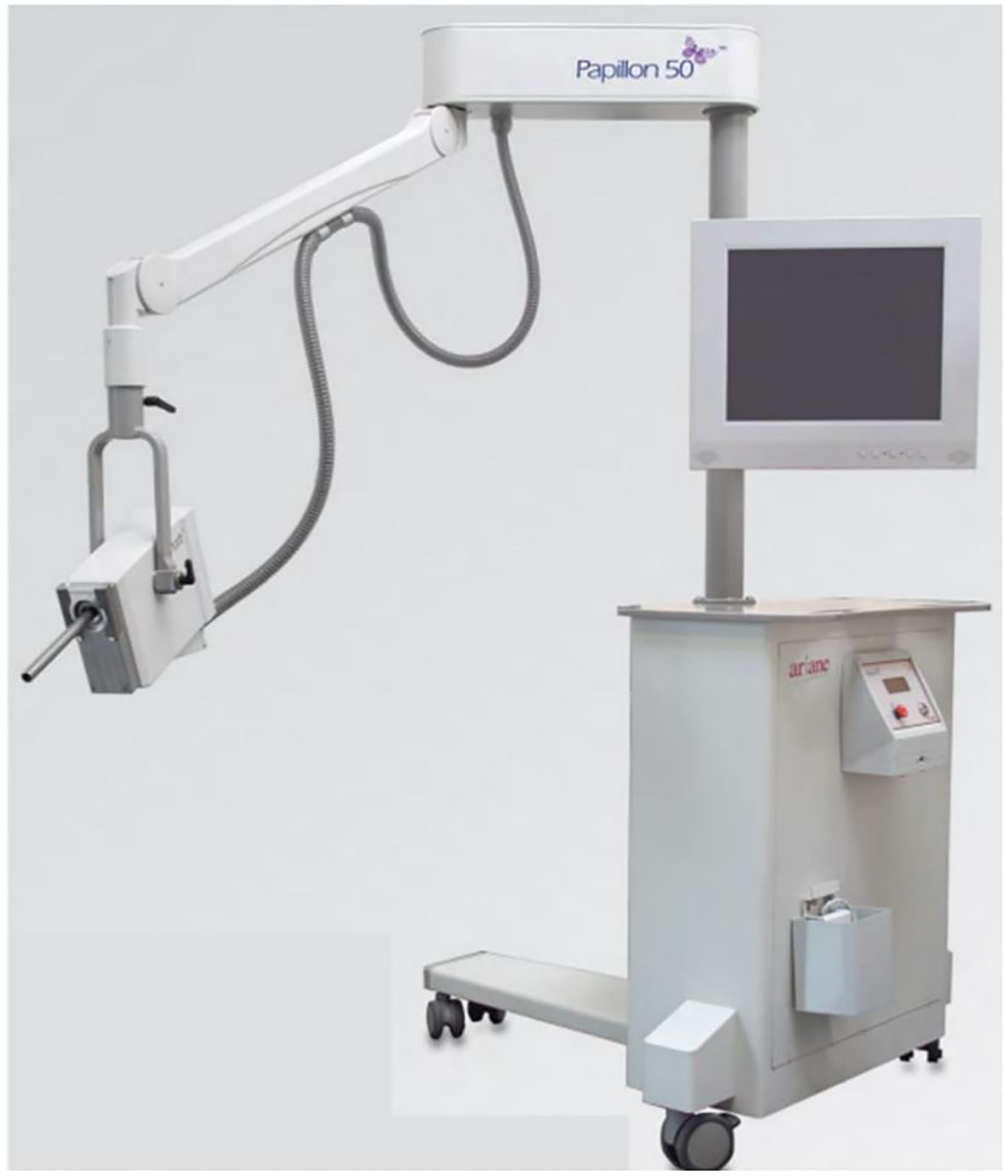
The Papillon 50™ contact X-ray brachytherapy system, equipped with a low­energy X-ray tube and interchangeable applicators of various diameters. The compact design allows outpatient delivery of high surface doses for rectal tumors.

**Figure 2:**
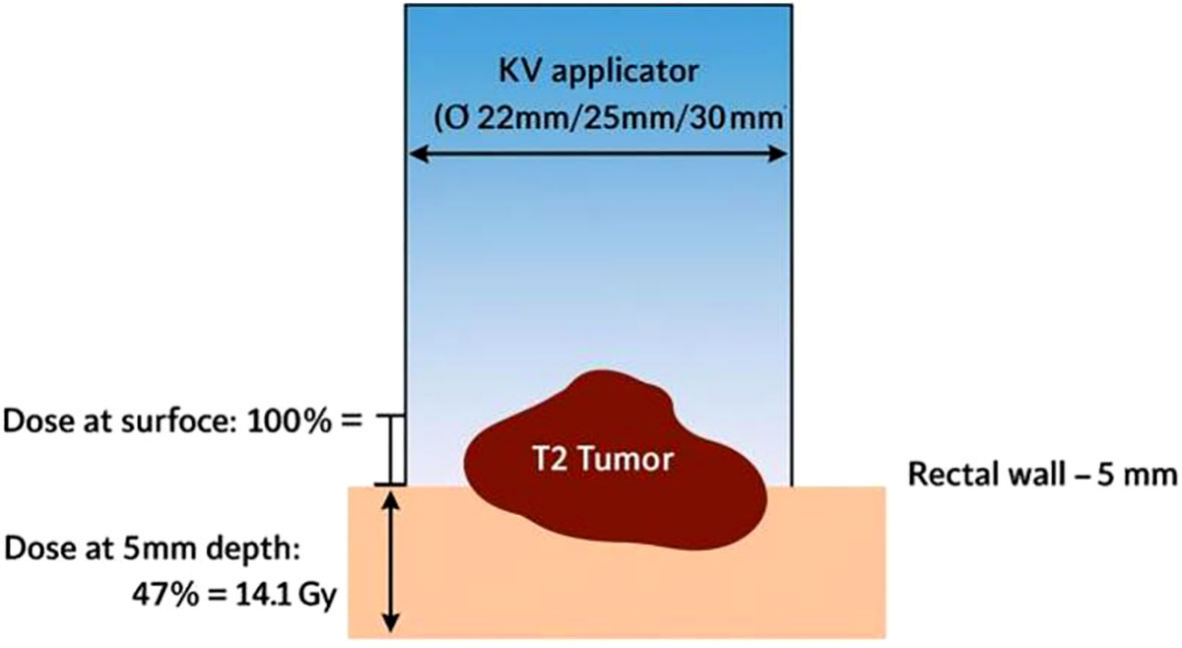
Schematic representation of Papillon treatment and dose distribution.

The Papillon CXB system consists of a compact, portable X-ray tube paired with a set of fixed-size applicators, enabling precise contact with the tumor and highly localized dose delivery (**Figure 1**). It uses low-energy X-rays (50 kV, HVL 0.53 mm Al, 2.7 mA), which generate a steep dose gradient that effectively spares surrounding healthy tissues (**Figure 2**). Applicator diameters range from 22 to 30 mm and are selected based on tumor size and location to ensure optimal fit and coverage. Treatments typically last 90 seconds to 3 minutes per session, delivering up to 30 Gy to the tumor surface.

**Figure 3:**
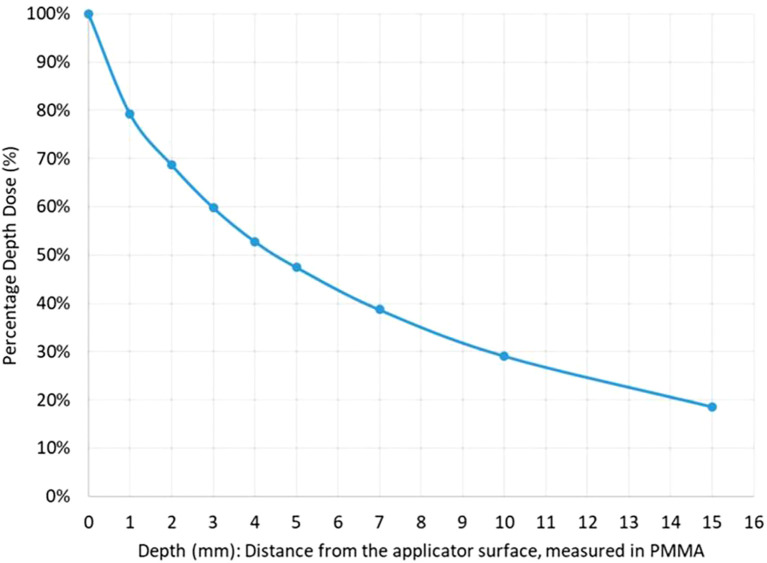
Dose fall-off curve. Depth-dose distribution curve measured in PMMA (polymethyl methacrylate), showing a rapid decline in radiation intensity. The dose is reduced by 50% at 4.4 mm from the applicator surface, underscoring the highly localized nature of CXB.

Due to the rapid dose fall-off, therapeutic radiation is essentially confined to the superficial tumor layers, with the dose halving at a depth of just 4.4 mm in PMMA (polymethyl methacrylate, a soft-tissue equivalent material used in dosimetric measurements) (**Figure 3**). Consequently, the high-dose region is limited to a small volume, only a few cubic centimeters within the applicator, ensuring that radiation remains concentrated at the tumor surface while sparing adjacent normal tissues and minimizing treatment-related toxicity.

In locally advanced cases, Papillon CXB is often used after chemoradiotherapy or total neoadjuvant therapy (TNT) as a targeted dose escalation or boost, usually in 3–4 weekly fractions, achieving a total biologically effective dose (BED) of around 300 Gy. For early-stage tumors (cT1–cT2), 2–3 fractions may suffice, depending on real-time clinical response.

Historically, the procedure was performed in the knee-chest position to facilitate access to anterior lesions. However, this position often caused discomfort, reducing patient cooperation and increasing movement. The gynecological position offers better tolerance, particularly for posterior lesions, but can lead to rectal collapse and impaired visualization. Additionally, introducing the applicator through the anal sphincter can be painful, especially with larger sizes, and attempts to mitigate this with local anesthesia have shown limited success. Discomfort is further amplified by the intimate nature of the procedure, which may provoke embarrassment and involuntary movements, affecting precision.

These factors led to the adoption of a refined Swiss protocol, integrating sedation and improved positioning to overcome these limitations and ensure patient comfort, applicator stability, and procedural accuracy.

## Indications for Papillon CXB

Papillon CXB is a versatile, patient-centered technique that supports a range of clinical scenarios in rectal cancer management. Its unique ability to deliver high-dose, localized radiation makes it particularly valuable in organ preservation strategies, without compromising oncologic control.

In early rectal cancers, CXB is used to maximize local control and preserve organ function. For small tumors (<3 cm), CXB is applied upfront before chemoradiotherapy, clearly enhancing response rates and improving long-term outcomes. In larger lesions, CXB serves as a post-CRT boost, consolidating downstaging and increasing the likelihood of achieving a complete clinical response (cCR), thereby avoiding radical surgery.

In locally advanced tumors requiring TNT with CRT, CXB provides an effective, targeted boost following downstaging. Its role in these regimens is being further explored in the ongoing TRESOR trial, with early results suggesting promising outcomes in terms of tumor control and organ preservation (20, 21).

Another increasingly relevant indication is the post-endoscopic resection of malignant polyps, especially in the lower rectum. In these cases, CXB targets residual microscopic disease at the resection site, offering a non-invasive alternative to radical surgery or external beam radiotherapy. This is particularly valuable for early-stage tumors located near the anorectal junction, where surgical intervention often results in high morbidity and a significant risk of permanent stoma. According to UK data, the permanent stoma rate following radical surgery in such cases can reach 32% (22). CXB provides a less invasive option, preserving quality of life without compromising local control.

Although primarily associated with non-operative management, CXB can also enhance surgical outcomes. When used preoperatively, it contributes to further tumor downstaging, improving the chances of sphincter preservation and reducing the need for permanent stomas. The Lyon R96–02 trial confirmed a durable functional benefit, with stoma rates reduced from 63% to 29% at 10 years in patients receiving CXB plus CRT.

Finally, CXB can also be employed for palliative intent, providing rapid symptom relief, such as bleeding, obstruction, or pain, in patients who are no longer candidates for curative treatment.

## Papillon treatment protocol in Switzerland

**Figure 4:**
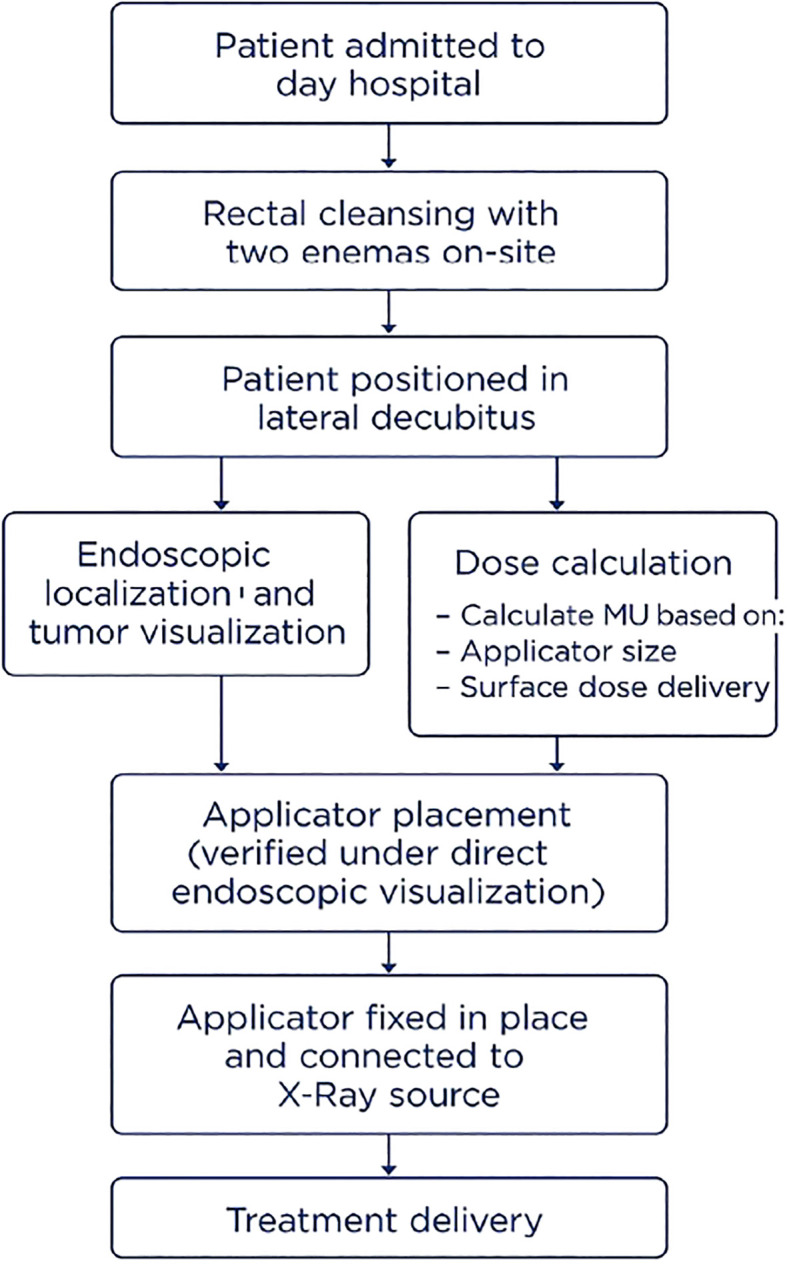
Flow chart of the Papillon CXB treatment protocol in Switzerland, outlining patient preparation, applicator placement, and treatment delivery.

To optimize patient outcomes and address traditional limitations, a streamlined protocol was developed, integrating gastroenterology expertise and a collaborative approach. This is summarized in the flow chart shown in **Figure 4**.

## Preparation and positioning

Patients are admitted to a day hospital setting, eliminating the need for complex home preparation. Rectal cleansing is standardized with two enemas administered on-site to ensure a clear distal bowel. Unlike traditional knee-chest or gynecological positioning, patients are placed in the lateral decubitus position (left or right, depending on tumor location). This position, inspired by colonoscopy practices, improves accessibility to anterior and posterior lesions while enhancing patient comfort, minimizing physical strain, and reducing the risk of involuntary movements.

## Sedation and patient comfort

Procedural sedation with propofol is administered by an anesthesiology or gastroenterology team to ensure patient stability and alleviate discomfort. This approach allows patients to maintain spontaneous breathing while relaxing the anal sphincter, facilitating smoother applicator insertion with minimal pain.

## Gastroenterologists involvement

A gastroenterologist plays a key role in optimizing the precision and effectiveness of Papillon CXB, particularly in technically demanding cases. An immediate pre-procedural rectosigmoidoscopy is performed to accurately localize the tumor. Endoscopic visualization enables irrigation and suction to clear residual fecal material, a common cause of poor visibility during CXB. Once the lesion is clearly exposed, the gastroenterologist assists in guiding and positioning the CXB applicator directly onto the target. This collaboration becomes especially important when treating patients after endoscopic resection of a malignant polyp, an indication that presents unique challenges. Without a visible tumor mass, the target area can be difficult to identify, and applicator positioning becomes less stable due to the lack of anatomical bulk. The gastroenterologist helps localize the scar or subtle mucosal changes and, when needed, marks the area using endoscopic clips or light cautery. These reference points improve visual orientation and allow for consistent, accurate applicator placement throughout the treatment course.

## Applicator placement and stability

**Figure 5:**
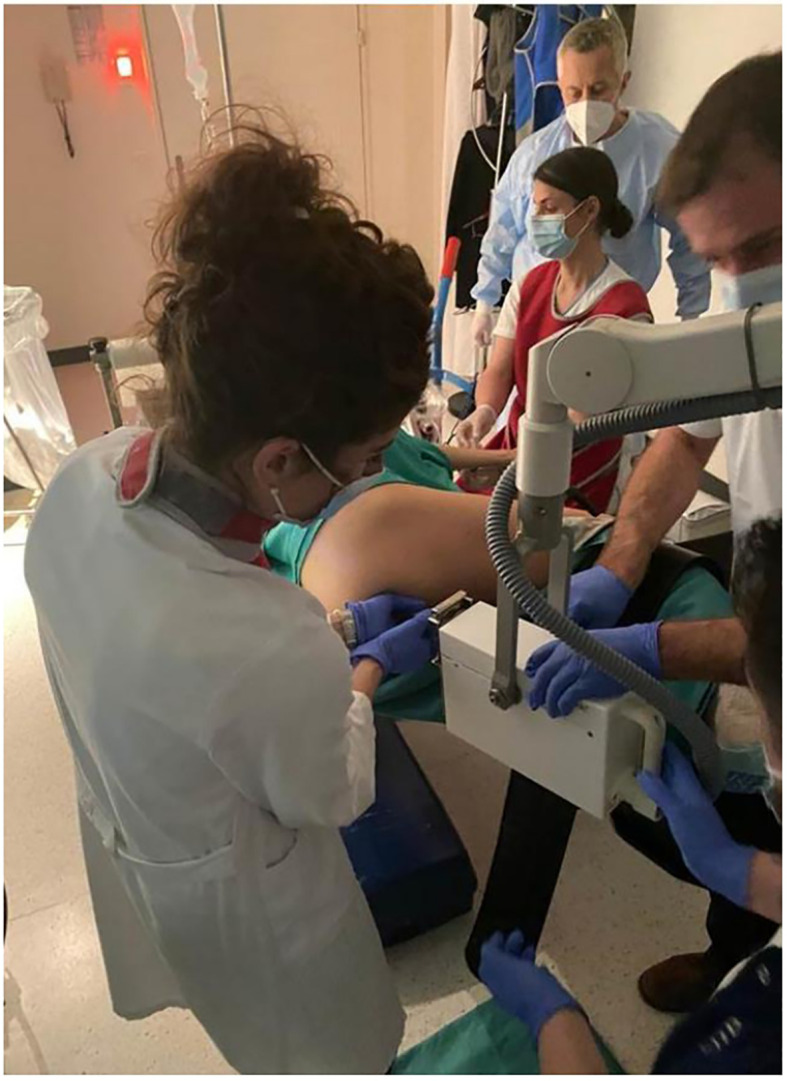
An example of papillon treatment in Switzerland with the patient positioned and sedated in the lateral position. In the figure are the radiation oncologist, the gastroenterologist with the nurse, and the radiation oncology technician.

The applicator’s position is carefully verified under direct endoscopic visualization to ensure optimal contact with the tumor. Once stability is confirmed, the applicator is securely fixed in place and connected to the X-ray source for treatment delivery. An exemple of applicator positioning during treatment is shown in **Figure 5**.

## Dose calculation in the Papillon technique

In the Papillon technique for contact radiotherapy, a simulation CT scan is not required because the treatment is delivered directly to the tumor surface, and the dose is calculated based on well-established physical parameters rather than 3D imaging.

The dose calculation is performed in parallel by the physicist or radiotherapy technician while the radiation oncologist and gastroenterologist position the applicator. This ensures that, once placement is confirmed, treatment can proceed without delay.

Dose prescription and delivery are standardized according to the applicator used and the monitor units delivered, based on calibration of machine output and applicator size. The calculation follows a simple, validated formula: MU = prescribed dose/(absolute dose factor × applicator factor.

## Safety and dosimetry

Radiation protection measures during Papillon CXB procedures comply with Swiss federal regulations. Given the low energy of the X-rays (50 kV), shielding requirements are minimal, and a dedicated bunker, such as those used for external beam radiotherapy, is not required. During beam delivery, staff either remain at the patient’s head, wearing protective lead aprons and thyroid collars, or stay behind a mobile radiation shield specifically designed for low-kV procedures. Occupational exposure is routinely monitored using ring and whole-body dosimeters to ensure adherence to dose limits.

## Discussion

The Swiss protocol for Papillon CXB represents a refined advancement in contact X-ray brachytherapy, successfully addressing two major procedural challenges: patient discomfort and applicator instability. Traditionally, treatments using the knee-chest or gynecological positions with minimal sedation, while clinically effective, often led to discomfort, movement-related issues, and reduced precision, especially in lower rectal tumors.

To overcome these limitations, early efforts included relocating the procedure to the operating room, where general anesthesia allowed for improved patient positioning and stability. However, this approach proved logistically complex, requiring operating room availability, anesthesiology support, and short hospital stays, making it impractical for routine outpatient use.

The current protocol integrates procedural sedation with propofol, lateral decubitus positioning, and close collaboration with gastroenterologists. The use of propofol sedation consistently alleviated sphincter spasms and anxiety, ensuring smoother applicator placement and improving overall tolerance of the procedure. Among the 73 patients treated with this approach, none reported severe pain, and no treatments were discontinued due to discomfort.

The applicator demonstrated excellent stability, maintaining consistent and accurate contact with the lesion in the majority of cases. Minor displacements were rarely observed in tumors located within three to four centimeters of the anal verge, but these were quickly corrected without affecting treatment quality.

Gastroenterologist involvement further enhanced accuracy, particularly when treating post-polypectomy scars or tumors in difficult-to-visualize locations. Pre-treatment endoscopic assessment ensured optimal visualization, facilitated cleaning of the rectal lumen, and enabled precise applicator positioning. This was sometimes assisted by endoscopic clips or cautery markings to define the target. This multidisciplinary approach not only improves technical accuracy but also fosters patient trust and comfort, addressing both physical and emotional challenges of rectal cancer treatment.

The procedural workflow was streamlined within a day hospital setting, with the entire process including preparation, sedation, positioning, and delivery completed in a median of 60 minutes. This optimized workflow minimized repeated attempts at applicator placement and reduced overall treatment time. Dosimetry consistently showed negligible occupational radiation exposure for staff.

Our previously published clinical data confirm that Papillon CXB achieves oncologic outcomes comparable to international benchmarks. While clinical effectiveness has already been demonstrated, the present protocol focuses on improving patient comfort, procedural accuracy, and feasibility in everyday clinical practice.

These procedural refinements have made Papillon CXB more reproducible, efficient, and patient-friendly, supporting its broader integration into rectal cancer care. While ongoing innovation such as improved applicator ergonomics or image-guided positioning may further enhance precision, the current protocol already offers a robust and scalable model for real-world implementation across institutions.

## Conclusion

Papillon contact X-ray brachytherapy (CXB) has demonstrated improved tumor response and higher organ preservation rates within organ-preserving strategies for rectal cancer.

By addressing common limitations of traditional CXB delivery, such as patient discomfort, applicator instability, and suboptimal visualization in anatomically challenging cases, this updated workflow enhances the technique through the integration of short-acting sedation, lateral decubitus positioning, and gastroenterologist-guided endoscopic placement. Our experience has shown improved patient tolerance and greater procedural accuracy, making this approach feasible and efficient within routine clinical practice.

As organ preservation becomes an increasingly central goal in rectal cancer management, we believe that the use of Papillon CXB will continue to expand. This refined protocol may serve as a practical model for other institutions aiming to implement or optimize CXB within their own organ-preserving treatment strategies.

## Data Availability

The raw data supporting the conclusions of this article will be made available by the authors, without undue reservation.
